# From Medical Imaging to Radiomics: Role of Data Science for Advancing Precision Health

**DOI:** 10.3390/jpm10010015

**Published:** 2020-03-02

**Authors:** Enrico Capobianco, Marco Dominietto

**Affiliations:** 1 Center for Computational Science, University of Miami, FL 33146, USA; 2 Paul Scherrer Institute, 5232 Villigen, Switzerland; marco-diego.dominietto@psi.ch

**Keywords:** precision health, medical imaging, radiomics, computational solutions, harmonization, modeling, validation

## Abstract

Treating disease according to precision health requires the individualization of therapeutic solutions as a cardinal step that is part of a process that typically depends on multiple factors. The starting point is the collection and assembly of data over time to assess the patient’s health status and monitor response to therapy. Radiomics is a very important component of this process. Its main goal is implementing a protocol to quantify the image informative contents by first mining and then extracting the most representative features. Further analysis aims to detect potential disease phenotypes through signs and marks of heterogeneity. As multimodal images hinge on various data sources, and these can be integrated with treatment plans and follow-up information, radiomics is naturally centered on dynamically monitoring disease progression and/or the health trajectory of patients. However, radiomics creates critical needs too. A concise list includes: (a) successful harmonization of intra/inter-modality radiomic measurements to facilitate the association with other data domains (genetic, clinical, lifestyle aspects, etc.); (b) ability of data science to revise model strategies and analytics tools to tackle multiple data types and structures (electronic medical records, personal histories, hospitalization data, genomic from various specimens, imaging, etc.) and to offer data-agnostic solutions for patient outcomes prediction; (c) and model validation with independent datasets to ensure generalization of results, clinical value of new risk stratifications, and support to clinical decisions for highly individualized patient management.

## 1. Introduction

Radiomics is generally associated with artificial intelligence (AI)- and machine learning (ML)-specific medical literature and embraces studies involving all medical imaging modalities. A search on PubMed (https://www.ncbi.nlm.nih.gov/pubmed) with the term “Radiomics” yields about 1450 publications. Most studies refer to oncological applications. For instance, O’ Connor reports the following important paragraph for defining radiomics: *“Radiomics uses computer algorithms to process the data collected by different medical imaging techniques and is becoming increasingly popular in cancer imaging research. One of its key characteristics is that radiomics recognizes that digital medical images are not only pictures—they are also are complex data. Radiomic analyses extract and measure an array of ‘features’ that describe the image texture and distributions of individual voxel values—the units that make up a 3D image—within a tumor. Each voxel represents a tiny amount of tissue and contains around 10^5^ to 10^7^ neoplastic and stromal cells, depending on tumor type and voxel dimensions.”* [1]. Also non-oncological studies have started to appear (examples include recent studies on cognitive disorders [2], neuron degeneration [3], cognitive impairment [4], hippocampal sclerosis [5], central nervous system [6], heart failure [7], stroke [8], postpartum haemorrhage [9], and carpal and cubital tunnel syndrome [10]).

Focusing on cancer and the different phenotypes that human cancers can select, our special interest goes to those that can be visualized non-invasively by *medical imaging* (MIM). Despite MIM increasing relevance in the clinical practice, combing its outcomes with those derived from genetic, clinical, and prognostic information is not routinely performed in the clinics. A knowledge gap therefore can be associated with partially unrevealed or yet unexplored evidence that such data could yield. A change in such direction is offered by *radiomics* (RAD) (see [11,12,13,14] among others). Appearing only a few years ago, it proposes a quantitative approach to capture value from images by features. These may either provide training sets for a variety of automated learning algorithms or be used to empower methods with robustness in both diagnosis and outcome prediction following specific treatment.

Computer-aided medical imaging (CAD-MIM) addressing diagnostics or therapy problems is not a new concept (see for instance [15,16,17]). CAD-MIM may first converge to a RAD feature space by providing evidence for clinical use through ad hoc image acquisition protocols and display methods, among others. Then, it might combine the quantitative measurements of the features into diagnostic scores in an attempt to extract disease-associated biomarkers.

However, the focus has now shifted to the design of integrated solutions able to offer support to clinical decisions by either software tools or data-driven analyses. The computational data science community has recently identified in RAD a rich application field for *deep learning* (DL) applications or a general ideal ground for employing ML and statistical methods, ensemble models (EM), visualization, and big data analytics. RAD’s greatest expected impact is of course expected at the medical community level, due to its association with clinical endpoints. Ultimately, every individual generates a health trajectory that reflects the individualized risk profile and/or responds differently to therapy, thus requiring personalized intervention as well as monitoring over time.

RAD work has stimulated research on the complex relationships among cancer biology, genomics, and clinical factors [18,19,20,21]. Three main computational factors have characterized the current scenario: (1) *The diminished criticality of the scale factor*: it is now possible to manage data volume, ambience, and breadth at unprecedented magnitudes. (2) *The increase of depth or resolution:* it is possible to reveal complex hierarchies of efficiently detected image details. (3) *The capacity to learn, validate, and generalize:* this happens at the systems level and translationally across context domains. These factors have contributed to the improvement of both contextualization and interpretation of clinically relevant data patterns, thus supporting the decision-making processes and increasing the potential of transforming clinical practice.

Support for radiation oncologists is especially necessary for decisions about individual treatments and disease prediction, thus defining patients’ prognostic paths. As anticipated, RAD leverages a two-step process to predict treatment outcome: (1) extraction of features, and (2) incorporation of the features into a computational model for predicting success/failure of therapy (even personalized therapy, as the ultimate goal). This process requires a dynamic model framework in order to perform integrated analysis of multiple data types that are recorded along the patient outcome trajectory (POT). While providing better discrimination between tumor phenotypes, the knowledge obtained from modeling integrated data will allow superior predictive power. DL or EM solutions are designed to coordinate and synthesize various types of measurements and predictions, respectively assembled and distributed across layers or reconciled into scores for more objective assessment and quantification of outcomes and endpoints. The dynamic value assigned to such models depends on their ability to perform integrated analysis of multiple data types recorded along the POT or the prognostic paths, and to predictively learn.

RAD requires deep assimilation by the actively involved research communities but appears inherently designed to add value in terms of precision. This in turn translates into the need of establishing more objective data interpretation standards and benchmarks. Both reference data and standardized methodologies must aim at the highest possible reproducibility and generalizability, including development across multiple centers and testing in prospective clinical trials. Currently, routine imaging technologies are employed in clinical trial settings largely centered on anatomic evaluations (size-based imaging endpoints) with very limited correlation with physiological outcome or early information about response to specific targeted therapies or pursuit of exploratory endpoints toward the design of the so-called integral cancer markers. Such limitations are in part induced by infrastructural changes involving imaging technologies or combination of imaging modalities, but also involve gaps in data collection strategies, digitalization, and other factors.

## 2. Imaging Biomarkers in Personalized Medicine

Metrics of performance involve both diagnostic and prognostic evaluations. MIM enables the non-invasive investigation of disease status, including temporal assessments of progression (i.e., disease trajectories). In many complex diseases, it is crucial to define response to treatment at early points in time, as this means there is the ability to determine the best possible prognostic path for the patient. Once specific targeted therapies are enabled, patient stratifications are expected by using the measured biomarker expression values.

*Imaging biomarkers* (IB), also those potentially outsourced by RAD, may represent spatio-temporal heterogeneity in cancer [22,23,24,25,26]. In principle, they offer a valuable and timely evaluation of patient outcomes while optimizing the trade-off between search of effective therapies and limitations of adverse effects. Highly recommended in patient management because they are minimally (or non) invasive, IB are direct expressions of bio-signals extracted from various examinations performed upon the patient. Also, IB represent unique expressions of disease phenotypes that need to be examined to establish correlation with other patients’ clinical data and known biological biomarkers. In such regards, radiomic procedures call for harmonization as an essential step to guarantee the reproducibility of the extracted features and the application of IB in multiple diagnostic contexts [27,28].

Structural, physiological, and molecular imaging can be considered all relevant for the above scopes. Structural imaging concerns morphological features related to tumor anatomy, whereas physiological imaging takes into consideration measures of fluid diffusion, perfusion, oxygenation, pH value, and microenvironment conditions at the tissue level. The molecular IB instead provide information regarding biological processes so as to assess heterogeneity and distinguish between malignant cells, specific cell populations, and other relevant factors.

## 3. Imaging Biobanking

Clearly enough, IB development is destined to proceed in parallel with the growth of the imaging biobanking field [29,30,31]. Here, diagnostic multimodal images generated by various technologies will be increasingly exploited by high-throughput computing to extract radiomic features, although translational gaps remain to be dealt with before achieving validation in clinical settings. Imaging biobanks linked to biological samples, omics, and patients’ clinical information are a new frontier whose final step is the next generation of integrated biobanks in which radiomic data will be merged with genomic, proteomic, or metabolomic findings in order to enable innovative and personalized approaches to disease treatment [32,33,34].

In such scenario, data science research would be focused to assemble a first layer of raw and processed data, metadata, combined feature measurements, and biomarkers derived from medical images, with another layer fueled by other disease-related factors such as patient prognosis, pathological findings, and genomic profiling. The products of this complex merge of organized, harmonized, and integrated data should become a genotype–phenotype-enriched system that is linkable to other repositories and subjected to full technical validation and further qualification for best possible routine clinical use [35]. Correspondingly, a final goal should consider assimilating such multilevel information and organize it into a prediction model with selected key features depending on defined clinical endpoints. Although validation is the necessary step to be covered to ensure clinical applicability of the model predictions, the used models could still require calibration in order to measure the matching of predictions with the observed clinical outcomes.

## 4. Image-to-Data Science Driven Research

It is reasonable to think that the wider the spectrum of imaging modalities is, and the more diverse the generated IB can be, then the higher the need of harmonization becomes. Reference goes to large-scale initiatives for measuring RAD contributions to high-quality standardized clinical care, such as The Cancer Imaging Archive (TCIA) (https://www.cancerimagingarchive.net/), which de-identifies and hosts a large archive of medical cancer images that are publicly accessible.

Then other important examples are provided by the Quantitative Imaging Network (QIN) (https://imaging.cancer.gov/programs_resources/specialized_initiatives/qin/about/default.htm), the Quantitative Imaging Biomarkers Alliance (QIBA) (https://www.rsna.org/en/research/quantitative-imaging-biomarkers-alliance), and the Image Biomarker Standardisation Initiative (IBSI) (https://arxiv.org/abs/1612.07003), with the latter being focused on reproducibility aspects in RAD studies.

Overall, and in light of the above initiatives, data science is totally central to RAD processes, from acquisition and assimilation of diverse data to feature extraction and selection for knowledge discovery until modeling, with the inclusion of both validation and calibration phases. In this scenario, the paradigm of clinical research is revised. Imaging is not just instrumental to prove a clinical hypothesis from evidence, but is also a tool generating quantitative data to be explained by models. To be interpretable by clinicians, data-driven results must respond to specific objectives identifying medical needs. For instance, MIM with multiple data modalities needs to be primarily assessed in its capacity of yielding complementary information of relevance to patients’ health and covering different data ranges, annotations, and spatial/temporal matching with multiomic data. The practical utility can be referred to the cancer field, in which cancer types differ substantially with regard to reference datasets and clinical/molecular annotations.

From a modeling standpoint, two main developments are currently extremely useful: *transfer learning* (TL) [36,37,38,39,40,41,42,43] and ensemble modeling (EM) approaches (see general introduction to the topic in [44]). TL assumes that the features learned in a certain application domain can be usefully applied to a different related domain. As this methodology aims at solving problems by leveraging solutions adopted in quite similar problems, it is natural to test the feature set ranking for consistency (across domains) and robustness (significance). Moreover, TL can intrinsically leverage similarity that may be hardly hypothesized by conventional approaches. EM is a reasonable strategy of weighting scores or predictions obtained from different models applied over similar or different datasets. The main rationale for using EM is reconciling evidence that otherwise could not be sufficiently informative at the individual level.

## 5. Radiomic Profiles: Construction and Interpretation

**Figure 1:**
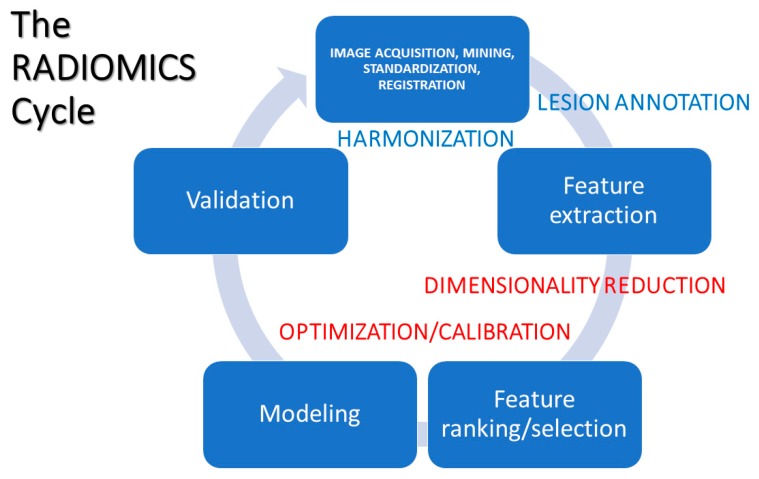
Typical steps in the radiomics (RAD) process.

RAD incorporates a variety of computational methods oriented to solve clinical problems, and usually focusing on analyses targeted to identify disease phenotypes. Ideally, such phenotypes should be correlated with other types of information from clinical reports, electronic health records, treatment response, genomic/proteomic/metabolomic assays, and other forms of information to detect biomarkers and quantitatively support decisions. Building radiomic profiles involves a few stages (see Figure 1), summarized as:

### 5.1. Data Acquisition, Mining, and Assimilation

Data collected from different sites, centers, and imaging modalities present different characteristics, parameters, and protocols that may influence the quality, comparability, and reproducibility of results.

### 5.2. Data Pre-Processing

This is usually aimed at reducing the variability and improving the robustness of the radiomic data features of interest. Here, the main scope is to mine the data and clean them from inconsistencies and errors that might complicate the next steps.

### 5.3. Feature Extraction

Data features are the syntheses between the content of MIM and the clinical endpoints that must be extracted from the available data, in part empirically (mainly semantic features requiring expert view) and in part computationally (algorithmically or model-driven features). This second class implies use of a variety of tools from statistics to ML, among other tools.

### 5.4. Feature Ranking and/or Selection

One needs to focus on the features with the highest predictive value, usually a specified set. Redundancies and spurious correlations need to be eliminated to reduce the risk of overfitting with which a large number of features might imply.

### 5.5. Modeling

Suitable models should be designed to measure the feature prediction power toward clinical outcomes by choosing within a vast range of variably supervised options.

### 5.6. Validation

This is the last critical stage, which brings value that is usable in clinical work with patients. The models are first assessed in their various performance aspects, and then their calibration is investigated to optimize, often iteratively, the matching between clinical outcomes and model predictions.

The literature is rich of examples of such applications (see for instance [45] describing a RAD prognostic vector applied to ovarian cancer, [46] in their work applied to Parkinson’s disease, and many others). In conclusion, RAD profiles are extremely useful for patient screening, detection, and monitoring scopes. The most valuable gains can be summarized as follows: (a) repeatability over time, (b) cost-effective collection of evidence, (c) assessment of spatial heterogeneity at a larger extent compared to biopsies, (d) significance of disease hallmarks, (e) identification of drug targets and biomarker roadmaps, (f) merge between qualitative and quantitative features obtained from different modalities, and (g) facilitated clinical decision making between alternative treatments on the basis of efficient monitoring of disease progression. This list is destined to be influenced by ongoing changes in technologies and methodologies according to PM direction.

The six stages identified above are complemented by other important tasks, reported in Figure 1 as lesion annotation, harmonization, dimensionality reduction, and optimization/calibration. The first task, lesion annotation, relies on various existing algorithms targeted to characteristic types that are frequently covered (skin, lung, liver, etc.; see for instance [47] for an algorithmic application or [48] for general discussion). The problems arise with types that are not frequent or that present together. Harmonization is then needed in RAD, especially for purposes of clinical translation, which would require the extracted features to be reproducible. Dimensionality reduction and optimization are mainly computational tasks whose scopes are to maximize significance and performance aimed at the best possible interpretability of results.

## 6. From Images to Networks

**Figure 2:**
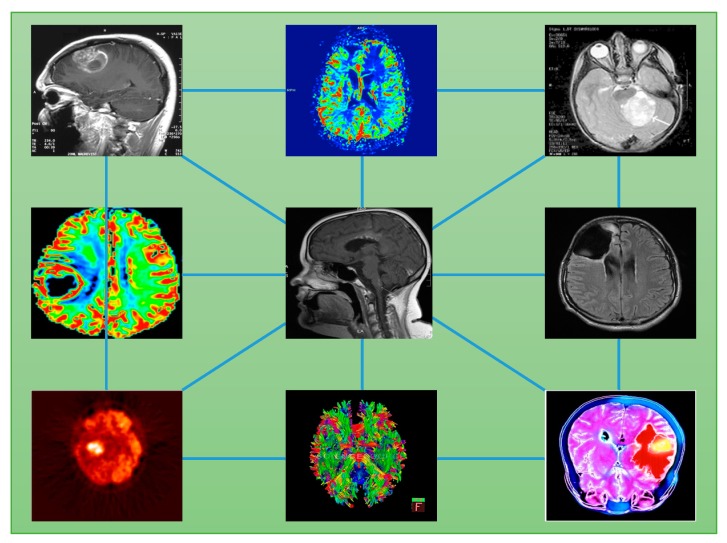
Image features integration from different imaging modalities.

Cancer heterogeneity implies complexity for which in-depth analysis is needed. The first consideration concerns the scale factor. Heterogeneity is present at various levels: genetic, molecular, cellular, and physiological, among others, which obviously leads to the search for a multiscale inference approach. This way, a blend of image readout information can be integrated into interpretable models as a mix of physiological properties such as perfusion, oxygenation, pH, and hypoxia; anatomical structure; histological characterization (necrotic, viable, angiogenic, etc.); molecular profile; phenotypic disease expression (intra-patient sub-clonality etc.); and more. Defining the stage of a tumor requires accounting for all such aspects, measuring and quantifying them in a way suitable to represent tumor states. However, data features gathered at a variety of spatio-temporal resolutions are hard to integrate into an interpretable predictive model (Figure 2) [49].

The level of sophistication achieved by imaging is relevant not only to the tumor but also to the host in an attempt to discover what changes are enabled in the presence of the former. Special emphasis goes naturally to the *host–tumor interface* (HTI) regions as the primary setting in which disease progression should be monitored because it is where mutual infiltration dynamics occur/from tumor to host and vice versa. These regions are highly heterogeneous also, as immune modulators, vessels, nutrients, and other typical factors depending on host interaction with tumor cells, proliferation factors, and angiogenic characteristics, among others. Another consideration goes to therapy, as treatment effects can be significantly measured in terms of tumor adaptation by evaluating HTI regions. Also, both distribution and frequency of targets for treatment might change during the disease course, implying that further heterogeneity needs to be considered at the clinical level [50].

**Figure 3:**
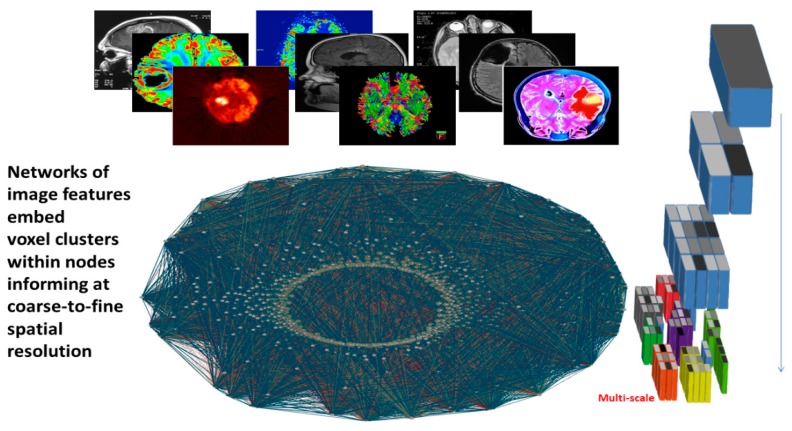
Multiscale information network processing from image features.

Networks are well-suited mathematical representations of complex data derived at different scales, as they engage nodes and links to quantify measurements in their evolving dynamics (Figure 3).

## 7. Impact Domains

**Figure 4:**
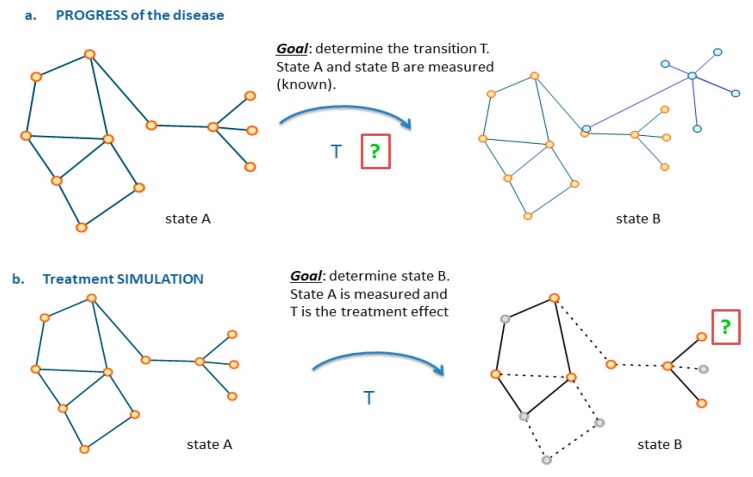
Use of networks to monitor disease progression (**a**) and simulate treatment (**b**). T is defined as general transition between states in the top panel, and as treatment (specifying the nature of transition) in the bottom panel. Two states are characterized, and the arrival state B is modified relatively to originating state A by the effect of T.

**Figure 5:**
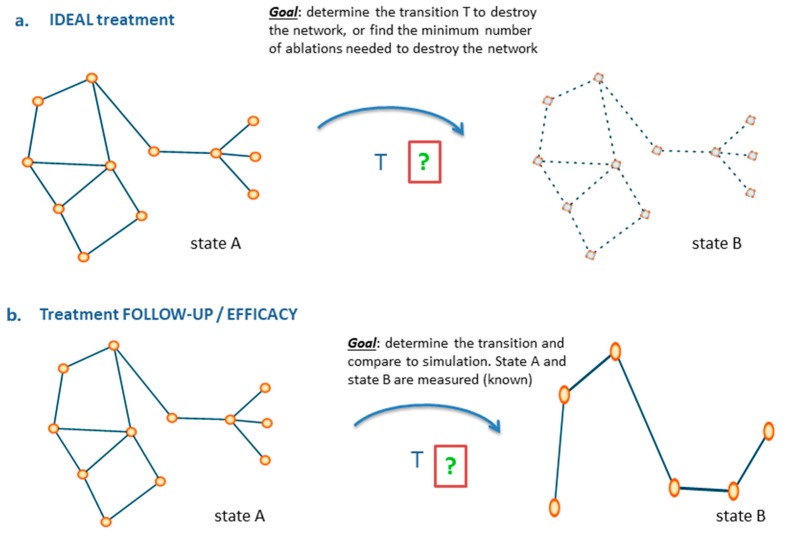
Use of networks to determine ideal treatment (**a**) and treatment follow-up (**b**). In the top panel the goal is to solve the tumor by acting on its nodes. Assuming that state A had defined the tumor nodes, state B measures the effects of ablations that destroy the communications between such nodes. The bottom panel measures the efficacy of treatment by tracking the shape of states (ex-ante and ex-post compared to treatment).

**Figure 6:**
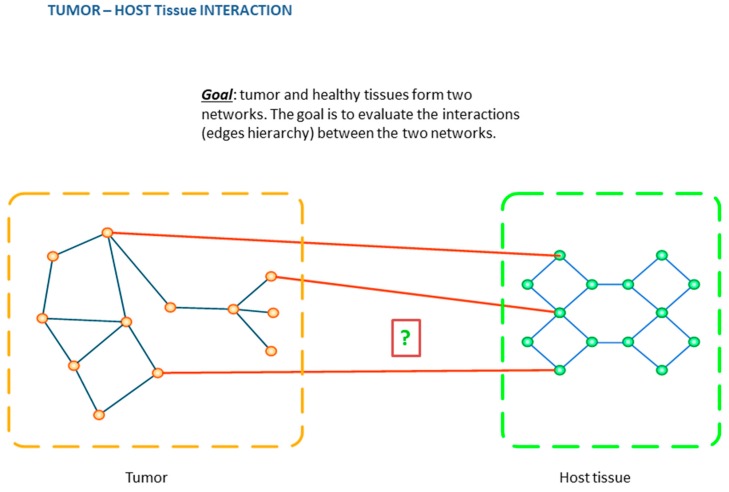
Use of networks to inspect tumor–host interaction dynamics. The network configurations in the two scenarios vary, with some common nodes that communicate differently.

Speaking of treatment and considering its effects over time (early response and follow-up), these can be monitored via networks by evaluating differential model configurations before and after treatment, as well as in cases determining what perturbation effects can be therapeutically induced to affect the HTI regions and/or destroy the tumor regions. Figure 4, Figure 5 and Figure 6 display states and state transitions with examples covering disease progression, treatment simulation, ideal treatment, treatment follow-up, and tumor–host interaction.

Although multiple factors can contribute to a non-uniform drug response, longitudinal heterogeneity refers to therapy-driven effects occurring over time, that is, along the disease course. These effects have been studied through moving targets between recurrent vs. primary GBM tumors [49]. Measuring target expression status over time and involving tissue at stage of primary and recurrent disease is in principle useful for diminishing the risk of incorrect treatment decisions. In practice, the underlying assumption is that functional relevance is assigned to measured expression values, which deserves cautious consideration due to other influences (epigenetic, lifestyle, etc.). However, a possible correlation between target expression and recurrent status of disease is not yet established on the basis of factors such as missing cutoff values, limited knowledge of whole extent of treatment response, and lack of reference predictive expression values.

Clinico-pathological heterogeneity has a strong molecular basis. Such heterogeneity prevents the identification of robust biomarkers through the identified gene signatures, for instance. Here, IB can be very useful. Different regions of the tumor may present different morphological features, a reason why pathologists evaluate multiple sections. Furthermore, spatial and temporal heterogeneity allows tumors to adapt to the microenvironment, and subclones tend to remain in a dynamic equilibrium to either compete or cooperate [51]. Functional and metabolic images can emphasize the critical role of tumor heterogeneity in clinics, as images provide non-invasive access to data arrays whose spatial information is obtained from individual voxels, that is, summaries of morphologic, metabolic, and physiologic information retrieved from a restricted tissue volume.

Quite evidently, there is a need to identify and measure spatial tumor heterogeneity to identify tumor subpopulations as responders or resistant with regards to therapy [26]. Imaging phenotypes may reflect histologic and genetic features that can to some extent correlate with patient outcome. The controversy of quantitative imaging for clinical use and decision support depends on the ability to design robust methods that are likely multidimensional and multiparametric. Taking single modalities might bring the risk of oversimplifying the complex dynamics under investigation (heterogeneity, metabolism) [52]. However, quantitative phenotypes of tumors (such as tumor size, shape, margin, and blood flow kinetics) have been already successfully associated with corresponding molecular profiles (DNA mutation, miRNA, protein, pathway gene expression, and copy number variation) (see [53]).

## 8. Concluding Remarks

Both precision and complexity are increasingly changing in the health research domain. These changes, already quite impressive in the imaging field, are destined to increase the clinical value of large-scale radiologic assessments and impact biomarker-based technologies in particular. More extended benchmarking of datasets will be requested, together with the inclusion of evidence from multiple modalities. An area of critical importance is clinical trials, in which imaging endpoints and IB need to be better defined and validated, respectively [54,55].

Together with inter-patient heterogeneity, also intra-patient heterogeneity is a source of uncertainty that requires in depth investigation. Therefore, a shift of focus toward variability imposes looking at the specific weight of both repeatability (repeated testing of the same subject over time) and reproducibility (measure of the same subject with different instruments of the same type) of measurements [56,57,58]. Reliability of RAD approaches depends on validating stable models, that is, those in which features have been thoroughly assessed for consistency and generalizability [59]. Data science and the associated computational machine learning tools are key enablers of quantification of spatially organized structures that for instance appear from imaging and characterize the tumor microenvironment.

Even if the application of sophisticated computational modeling tools is now part of the evolution of MIM and RAD fields, and translational aspects have become critical [60], two main issues that remain controversial still call for a solution. The first issue is a lack of a consensus on what learning method should be used or what feature selection should be operated (refer to IBSI, for instance). Although stable models should be more robust, the problem goes beyond modeling aspects and involves high-quality data and standardized processes [13]. The second issue is interpretability of results. It is reasonable to think that this allows us to trust or not trust the predictions obtained by the tools [61]. In such regards, emerging techniques promise to have wide impacts in the medical field, and in the realm of DL, specific reference goes to variational autoencoders [62] and generative adversarial networks [63], both able to learn large amounts of data in an unsupervised fashion (see [64] for more details). Linked to these tasks, there is also the investigation of strategies to combine imaging modalities to improve detection power and precision diagnostics [65].

In conclusion, radiomics reveals disease characteristics that are not simply visible from images alone. To yield useful measurement, researchers can, for instance, identify features highly predictive for time to progression and overall survival, as well as response to therapy. Such findings may help identify patients at low-to-high risk for disease progression and recurrence. The benefit of predicting in advance of therapy, to which patients are likely to respond or not, helps treatment remodulation for some patients (for instance, chemotherapy versus radiation or immunotherapy) or indicates need of relying on more intensive observation and follow-up for other patients.
